# Supplementary material for: Anisotropic Characterizations of Electrospun PAN Nanofiber Mats Using Design of Experiments
Source: Nanomaterials (Basel). 2020 Nov 17;10(11):2273. doi: 10.3390/nano10112273 (PMC7698451; doi:10.3390/nano10112273)
Supplement: Supplementary file 1 [file nanomaterials-10-02273-s001.zip › SI.docx]

Supplementary Materials

Anisotropic Characterizations of Electrospun PAN Nanofiber Mats Using Design of Experiments

Blesson Isaac ^1,^*, Robert M. Taylor ^2^ and Kenneth Reifsnider ^2^

^1^ Chemical and Radiation Measurement Department, Energy Environment Science and Technology, Idaho National Laboratory, Idaho Falls, ID 83415, USA

^2^ Department of Mechanical and Aerospace Engineering, The University of Texas at Arlington, Arlington, TX 76019, USA; taylorrm@uta.edu (R.M.T.); kenneth.reifsnider@uta.edu (K.R.)

***** Correspondence: blesson.isaac@inl.gov; Tel.: +1-713-553-4037

**Table S1.** Angular measuremnent of original system.

|  | Label | Area | Mean | Min | Max | Angle |
| --- | --- | --- | --- | --- | --- | --- |
| 1 | 8 w%-4 V_original.jpg | 0 | 0 | 0 | 0 | 73.487 |
| 2 | 8 w%-4 V_original.jpg | 0 | 0 | 0 | 0 | 172.999 |
| 3 | 8 w%-4 V_original.jpg | 0 | 0 | 0 | 0 | 88.698 |
| 4 | 8 w%-4 V_original.jpg | 0 | 0 | 0 | 0 | 11.459 |
| 5 | 8 w%-4 V_original.jpg | 0 | 0 | 0 | 0 | 7.8 |
| 6 | 8 w%-4 V_original.jpg | 0 | 0 | 0 | 0 | 119.291 |
| 7 | 8 w%-4 V_original.jpg | 0 | 0 | 0 | 0 | 7.496 |
| 8 | 8 w%-4 V_original.jpg | 0 | 0 | 0 | 0 | 35.311 |
| 9 | 8 w%-4 V_original.jpg | 0 | 0 | 0 | 0 | 156.038 |
| 10 | 8 w%-4 V_original.jpg | 0 | 0 | 0 | 0 | 70.641 |
| 11 | 8 w%-4 V_original.jpg | 0 | 0 | 0 | 0 | 123.275 |
| 12 | 8 w%-4 V_original.jpg | 0 | 0 | 0 | 0 | 131.496 |
| 13 | 8 w%-4 V_original.jpg | 0 | 0 | 0 | 0 | 0 |
| 14 | 8 w%-4 V_original.jpg | 0 | 0 | 0 | 0 | 19.179 |
| 15 | 8 w%-4 V_original.jpg | 0 | 0 | 0 | 0 | 173.093 |
| 16 | 8 w%-4 V_original.jpg | 0 | 0 | 0 | 0 | 38.418 |
| 17 | 8 w%-4 V_original.jpg | 0 | 0 | 0 | 0 | 51.072 |
| 18 | 8 w%-4 V_original.jpg | 0 | 0 | 0 | 0 | 65.225 |
| 19 | 8 w%-4 V_original.jpg | 0 | 0 | 0 | 0 | 112.109 |
| 20 | 8 w%-4 V_original.jpg | 0 | 0 | 0 | 0 | 8.259 |
| 21 | 8 w%-4 V_original.jpg | 0 | 0 | 0 | 0 | 13.736 |
| 22 | 8 w%-4 V_original.jpg | 0 | 0 | 0 | 0 | 49.764 |
| 23 | 8 w%-4 V_original.jpg | 0 | 0 | 0 | 0 | 75.964 |
| 24 | 8 w%-4 V_original.jpg | 0 | 0 | 0 | 0 | 46.637 |
| 25 | 8 w%-4 V_original.jpg | 0 | 0 | 0 | 0 | 24.624 |
| 26 | 8 w%-4 V_original.jpg | 0 | 0 | 0 | 0 | 1.061 |
| 27 | 8 w%-4 V_original.jpg | 0 | 0 | 0 | 0 | 66.194 |
| 28 | 8 w%-4 V_original.jpg | 0 | 0 | 0 | 0 | 17.592 |
| 29 | 8 w%-4 V_original.jpg | 0 | 0 | 0 | 0 | 155.433 |
| 30 | 8 w%-4 V_original.jpg | 0 | 0 | 0 | 0 | 145.008 |
| 31 | 8 w%-4 V_original.jpg | 0 | 0 | 0 | 0 | 162.072 |
| 32 | 8 w%-4 V_original.jpg | 0 | 0 | 0 | 0 | 34.439 |
| 33 | 8 w%-4 V_original.jpg | 0 | 0 | 0 | 0 | 90 |
| 34 | 8 w%-4 V_original.jpg | 0 | 0 | 0 | 0 | 8.531 |
| 35 | 8 w%-4 V_original.jpg | 0 | 0 | 0 | 0 | 101.592 |
| 36 | 8 w%-4 V_original.jpg | 0 | 0 | 0 | 0 | 62.241 |
| 37 | 8 w%-4 V_original.jpg | 0 | 0 | 0 | 0 | 53.471 |
| 38 | 8 w%-4 V_original.jpg | 0 | 0 | 0 | 0 | 26.565 |
| 39 | 8 w%-4 V_original.jpg | 0 | 0 | 0 | 0 | 128.047 |
| 40 | 8 w%-4 V_original.jpg | 0 | 0 | 0 | 0 | 51.34 |
| 41 | 8 w%-4 V_original.jpg | 0 | 0 | 0 | 0 | 142.001 |
| 42 | 8 w%-4 V_original.jpg | 0 | 0 | 0 | 0 | 45 |
| 43 | 8 w%-4 V_original.jpg | 0 | 0 | 0 | 0 | 163.301 |
| 44 | 8 w%-4 V_original.jpg | 0 | 0 | 0 | 0 | 30.7 |
| 45 | 8 w%-4 V_original.jpg | 0 | 0 | 0 | 0 | 55.176 |
| 46 | 8 w%-4 V_original.jpg | 0 | 0 | 0 | 0 | 21.318 |
| 47 | 8 w%-4 V_original.jpg | 0 | 0 | 0 | 0 | 58.671 |
| 48 | 8 w%-4 V_original.jpg | 0 | 0 | 0 | 0 | 50.194 |
| 49 | 8 w%-4 V_original.jpg | 0 | 0 | 0 | 0 | 131.634 |
| 50 | 8 w%-4 V_original.jpg | 0 | 0 | 0 | 0 | 16.557 |
| 51 | 8 w%-4 V_original.jpg | 0 | 0 | 0 | 0 | 90 |
| 52 | 8 w%-4 V_original.jpg | 0 | 0 | 0 | 0 | 102.995 |
| 53 | 8 w%-4 V_original.jpg | 0 | 0 | 0 | 0 | 169.624 |
| 54 | 8 w%-4 V_original.jpg | 0 | 0 | 0 | 0 | 162.3 |
| 55 | 8 w%-4 V_original.jpg | 0 | 0 | 0 | 0 | 11.889 |
| 56 | 8 w%-4 V_original.jpg | 0 | 0 | 0 | 0 | 37.747 |
| 57 | 8 w%-4 V_original.jpg | 0 | 0 | 0 | 0 | 11.47 |
| 58 | 8 w%-4 V_original.jpg | 0 | 0 | 0 | 0 | 31.535 |
| 59 | 8 w%-4 V_original.jpg | 0 | 0 | 0 | 0 | 40.101 |
| 60 | 8 w%-4 V_original.jpg | 0 | 0 | 0 | 0 | 142.509 |
| 61 | 8 w%-4 V_original.jpg | 0 | 0 | 0 | 0 | 26.565 |
| 62 | 8 w%-4 V_original.jpg | 0 | 0 | 0 | 0 | 60.068 |
| 63 | 8 w%-4 V_original.jpg | 0 | 0 | 0 | 0 | 18.138 |
| 64 | 8 w%-4 V_original.jpg | 0 | 0 | 0 | 0 | 158.429 |
| 65 | 8 w%-4 V_original.jpg | 0 | 0 | 0 | 0 | 173.66 |
| 66 | 8 w%-4 V_original.jpg | 0 | 0 | 0 | 0 | 123.977 |
| 67 | 8 w%-4 V_original.jpg | 0 | 0 | 0 | 0 | 23.629 |
| 68 | 8 w%-4 V_original.jpg | 0 | 0 | 0 | 0 | 53.947 |
| 69 | 8 w%-4 V_original.jpg | 0 | 0 | 0 | 0 | 9.752 |
| 70 | 8 w%-4 V_original.jpg | 0 | 0 | 0 | 0 | 68.199 |
| 71 | 8 w%-4 V_original.jpg | 0 | 0 | 0 | 0 | 64.179 |
| 72 | 8 w%-4 V_original.jpg | 0 | 0 | 0 | 0 | 14.744 |
| 73 | 8 w%-4 V_original.jpg | 0 | 0 | 0 | 0 | 14.534 |
| 74 | 8 w%-4 V_original.jpg | 0 | 0 | 0 | 0 | 176.309 |
| 75 | 8 w%-4 V_original.jpg | 0 | 0 | 0 | 0 | 167.775 |
| 76 | 8 w%-4 V_original.jpg | 0 | 0 | 0 | 0 | 128.29 |
| 77 | 8 w%-4 V_original.jpg | 0 | 0 | 0 | 0 | 81.87 |
| 78 | 8 w%-4 V_original.jpg | 0 | 0 | 0 | 0 | 154.592 |
| 79 | 8 w%-4 V_original.jpg | 0 | 0 | 0 | 0 | 146.31 |
| 80 | 8 w%-4 V_original.jpg | 0 | 0 | 0 | 0 | 25.942 |
| 81 | 8 w%-4 V_original.jpg | 0 | 0 | 0 | 0 | 55.491 |
| 82 | 8 w%-4 V_original.jpg | 0 | 0 | 0 | 0 | 112.109 |
| 83 | 8 w%-4 V_original.jpg | 0 | 0 | 0 | 0 | 45 |
| 84 | 8 w%-4 V_original.jpg | 0 | 0 | 0 | 0 | 32.905 |
| 85 | 8 w%-4 V_original.jpg | 0 | 0 | 0 | 0 | 28.443 |
| 86 | 8 w%-4 V_original.jpg | 0 | 0 | 0 | 0 | 30.256 |
| 87 | 8 w%-4 V_original.jpg | 0 | 0 | 0 | 0 | 170.538 |
| 88 | 8 w%-4 V_original.jpg | 0 | 0 | 0 | 0 | 166.13 |
| 89 | 8 w%-4 V_original.jpg | 0 | 0 | 0 | 0 | 16.144 |
| 90 | 8 w%-4 V_original.jpg | 0 | 0 | 0 | 0 | 163.034 |
| 91 | 8 w%-4 V_original.jpg | 0 | 0 | 0 | 0 | 168.826 |
| 92 | 8 w%-4 V_original.jpg | 0 | 0 | 0 | 0 | 60.068 |
| 93 | 8 w%-4 V_original.jpg | 0 | 0 | 0 | 0 | 60.751 |
| 94 | 8 w%-4 V_original.jpg | 0 | 0 | 0 | 0 | 166.464 |
| 95 | 8 w%-4 V_original.jpg | 0 | 0 | 0 | 0 | 160.346 |
| 96 | 8 w%-4 V_original.jpg | 0 | 0 | 0 | 0 | 18.06 |
| 97 | 8 w%-4 V_original.jpg | 0 | 0 | 0 | 0 | 16.699 |
| 98 | 8 w%-4 V_original.jpg | 0 | 0 | 0 | 0 | 66.644 |
| 99 | 8 w%-4 V_original.jpg | 0 | 0 | 0 | 0 | 9.019 |
| 100 | 8 w%-4 V_original.jpg | 0 | 0 | 0 | 0 | 10.008 |
| 101 | 8 w%-4 V_original.jpg | 0 | 0 | 0 | 0 | 63.435 |
| 102 | 8 w%-4 V_original.jpg | 0 | 0 | 0 | 0 | 59.349 |
| 103 | 8 w%-4 V_original.jpg | 0 | 0 | 0 | 0 | 16.699 |
| 104 | 8 w%-4 V_original.jpg | 0 | 0 | 0 | 0 | 144.866 |
| 105 | 8 w%-4 V_original.jpg | 0 | 0 | 0 | 0 | 150.461 |
| 106 | 8 w%-4 V_original.jpg | 0 | 0 | 0 | 0 | 45 |
| 107 | 8 w%-4 V_original.jpg | 0 | 0 | 0 | 0 | 167.471 |
| 108 | 8 w%-4 V_original.jpg | 0 | 0 | 0 | 0 | 155.095 |
| 109 | 8 w%-4 V_original.jpg | 0 | 0 | 0 | 0 | 46.397 |
| 110 | 8 w%-4 V_original.jpg | 0 | 0 | 0 | 0 | 175.601 |
|  |  |  |  |  | Mean | 77.87815 |
|  |  |  |  |  | Standard Deviation | 58.03588 |

**Table S2.** Angular measurement of improved system.

|  | Label | Area | Mean | Min | Max | Angle |
| --- | --- | --- | --- | --- | --- | --- |
| 1 | 8w-4v-improved.jpg | 0 | 0 | 0 | 0 | 23.806 |
| 2 | 8w-4v-improved.jpg | 0 | 0 | 0 | 0 | 63.435 |
| 3 | 8w-4v-improved.jpg | 0 | 0 | 0 | 0 | 20.956 |
| 4 | 8w-4v-improved.jpg | 0 | 0 | 0 | 0 | 145.491 |
| 5 | 8w-4v-improved.jpg | 0 | 0 | 0 | 0 | 163.202 |
| 6 | 8w-4v-improved.jpg | 0 | 0 | 0 | 0 | 19.799 |
| 7 | 8w-4v-improved.jpg | 0 | 0 | 0 | 0 | 62.904 |
| 8 | 8w-4v-improved.jpg | 0 | 0 | 0 | 0 | 59.744 |
| 9 | 8w-4v-improved.jpg | 0 | 0 | 0 | 0 | 121.608 |
| 10 | 8w-4v-improved.jpg | 0 | 0 | 0 | 0 | 23.429 |
| 11 | 8w-4v-improved.jpg | 0 | 0 | 0 | 0 | 39.56 |
| 12 | 8w-4v-improved.jpg | 0 | 0 | 0 | 0 | 38.66 |
| 13 | 8w-4v-improved.jpg | 0 | 0 | 0 | 0 | 32.276 |
| 14 | 8w-4v-improved.jpg | 0 | 0 | 0 | 0 | 49.399 |
| 15 | 8w-4v-improved.jpg | 0 | 0 | 0 | 0 | 93.468 |
| 16 | 8w-4v-improved.jpg | 0 | 0 | 0 | 0 | 58.831 |
| 17 | 8w-4v-improved.jpg | 0 | 0 | 0 | 0 | 176.634 |
| 18 | 8w-4v-improved.jpg | 0 | 0 | 0 | 0 | 20.433 |
| 19 | 8w-4v-improved.jpg | 0 | 0 | 0 | 0 | 50.389 |
| 20 | 8w-4v-improved.jpg | 0 | 0 | 0 | 0 | 168.179 |
| 21 | 8w-4v-improved.jpg | 0 | 0 | 0 | 0 | 167.093 |
| 22 | 8w-4v-improved.jpg | 0 | 0 | 0 | 0 | 31.608 |
| 23 | 8w-4v-improved.jpg | 0 | 0 | 0 | 0 | 29.427 |
| 24 | 8w-4v-improved.jpg | 0 | 0 | 0 | 0 | 105.945 |
| 25 | 8w-4v-improved.jpg | 0 | 0 | 0 | 0 | 136.848 |
| 26 | 8w-4v-improved.jpg | 0 | 0 | 0 | 0 | 115.463 |
| 27 | 8w-4v-improved.jpg | 0 | 0 | 0 | 0 | 120.579 |
| 28 | 8w-4v-improved.jpg | 0 | 0 | 0 | 0 | 115.201 |
| 29 | 8w-4v-improved.jpg | 0 | 0 | 0 | 0 | 35.134 |
| 30 | 8w-4v-improved.jpg | 0 | 0 | 0 | 0 | 46.637 |
| 31 | 8w-4v-improved.jpg | 0 | 0 | 0 | 0 | 158.962 |
| 32 | 8w-4v-improved.jpg | 0 | 0 | 0 | 0 | 103.241 |
| 33 | 8w-4v-improved.jpg | 0 | 0 | 0 | 0 | 38.367 |
| 34 | 8w-4v-improved.jpg | 0 | 0 | 0 | 0 | 54.293 |
| 35 | 8w-4v-improved.jpg | 0 | 0 | 0 | 0 | 36.87 |
| 36 | 8w-4v-improved.jpg | 0 | 0 | 0 | 0 | 43.152 |
| 37 | 8w-4v-improved.jpg | 0 | 0 | 0 | 0 | 69.444 |
| 38 | 8w-4v-improved.jpg | 0 | 0 | 0 | 0 | 135.939 |
| 39 | 8w-4v-improved.jpg | 0 | 0 | 0 | 0 | 25.201 |
| 40 | 8w-4v-improved.jpg | 0 | 0 | 0 | 0 | 139.635 |
| 41 | 8w-4v-improved.jpg | 0 | 0 | 0 | 0 | 140.711 |
| 42 | 8w-4v-improved.jpg | 0 | 0 | 0 | 0 | 74.932 |
| 43 | 8w-4v-improved.jpg | 0 | 0 | 0 | 0 | 32.905 |
| 44 | 8w-4v-improved.jpg | 0 | 0 | 0 | 0 | 38.66 |
| 45 | 8w-4v-improved.jpg | 0 | 0 | 0 | 0 | 22.249 |
| 46 | 8w-4v-improved.jpg | 0 | 0 | 0 | 0 | 45 |
| 47 | 8w-4v-improved.jpg | 0 | 0 | 0 | 0 | 95.44 |
| 48 | 8w-4v-improved.jpg | 0 | 0 | 0 | 0 | 13.627 |
| 49 | 8w-4v-improved.jpg | 0 | 0 | 0 | 0 | 160.974 |
| 50 | 8w-4v-improved.jpg | 0 | 0 | 0 | 0 | 104.036 |
| 51 | 8w-4v-improved.jpg | 0 | 0 | 0 | 0 | 11.31 |
| 52 | 8w-4v-improved.jpg | 0 | 0 | 0 | 0 | 31.329 |
| 53 | 8w-4v-improved.jpg | 0 | 0 | 0 | 0 | 55.981 |
| 54 | 8w-4v-improved.jpg | 0 | 0 | 0 | 0 | 157.068 |
| 55 | 8w-4v-improved.jpg | 0 | 0 | 0 | 0 | 70.936 |
| 56 | 8w-4v-improved.jpg | 0 | 0 | 0 | 0 | 120.069 |
| 57 | 8w-4v-improved.jpg | 0 | 0 | 0 | 0 | 110.136 |
| 58 | 8w-4v-improved.jpg | 0 | 0 | 0 | 0 | 5.856 |
| 59 | 8w-4v-improved.jpg | 0 | 0 | 0 | 0 | 4.844 |
| 60 | 8w-4v-improved.jpg | 0 | 0 | 0 | 0 | 5.492 |
| 61 | 8w-4v-improved.jpg | 0 | 0 | 0 | 0 | 148.392 |
| 62 | 8w-4v-improved.jpg | 0 | 0 | 0 | 0 | 58.671 |
| 63 | 8w-4v-improved.jpg | 0 | 0 | 0 | 0 | 131.76 |
| 64 | 8w-4v-improved.jpg | 0 | 0 | 0 | 0 | 117.759 |
| 65 | 8w-4v-improved.jpg | 0 | 0 | 0 | 0 | 34.592 |
| 66 | 8w-4v-improved.jpg | 0 | 0 | 0 | 0 | 151.189 |
| 67 | 8w-4v-improved.jpg | 0 | 0 | 0 | 0 | 172.235 |
| 68 | 8w-4v-improved.jpg | 0 | 0 | 0 | 0 | 55.176 |
| 69 | 8w-4v-improved.jpg | 0 | 0 | 0 | 0 | 25.641 |
| 70 | 8w-4v-improved.jpg | 0 | 0 | 0 | 0 | 5.877 |
| 71 | 8w-4v-improved.jpg | 0 | 0 | 0 | 0 | 29.745 |
| 72 | 8w-4v-improved.jpg | 0 | 0 | 0 | 0 | 73.443 |
| 73 | 8w-4v-improved.jpg | 0 | 0 | 0 | 0 | 174.987 |
| 74 | 8w-4v-improved.jpg | 0 | 0 | 0 | 0 | 68.199 |
| 75 | 8w-4v-improved.jpg | 0 | 0 | 0 | 0 | 69.59 |
| 76 | 8w-4v-improved.jpg | 0 | 0 | 0 | 0 | 102.724 |
| 77 | 8w-4v-improved.jpg | 0 | 0 | 0 | 0 | 13.134 |
| 78 | 8w-4v-improved.jpg | 0 | 0 | 0 | 0 | 106.26 |
| 79 | 8w-4v-improved.jpg | 0 | 0 | 0 | 0 | 125.538 |
| 80 | 8w-4v-improved.jpg | 0 | 0 | 0 | 0 | 14.534 |
| 81 | 8w-4v-improved.jpg | 0 | 0 | 0 | 0 | 173.333 |
| 82 | 8w-4v-improved.jpg | 0 | 0 | 0 | 0 | 19.537 |
| 83 | 8w-4v-improved.jpg | 0 | 0 | 0 | 0 | 11.976 |
| 84 | 8w-4v-improved.jpg | 0 | 0 | 0 | 0 | 104.47 |
| 85 | 8w-4v-improved.jpg | 0 | 0 | 0 | 0 | 138.814 |
| 86 | 8w-4v-improved.jpg | 0 | 0 | 0 | 0 | 52.431 |
| 87 | 8w-4v-improved.jpg | 0 | 0 | 0 | 0 | 8.455 |
| 88 | 8w-4v-improved.jpg | 0 | 0 | 0 | 0 | 166.373 |
| 89 | 8w-4v-improved.jpg | 0 | 0 | 0 | 0 | 130.914 |
| 90 | 8w-4v-improved.jpg | 0 | 0 | 0 | 0 | 110.376 |
| 91 | 8w-4v-improved.jpg | 0 | 0 | 0 | 0 | 145.305 |
| 92 | 8w-4v-improved.jpg | 0 | 0 | 0 | 0 | 63.435 |
| 93 | 8w-4v-improved.jpg | 0 | 0 | 0 | 0 | 113.429 |
| 94 | 8w-4v-improved.jpg | 0 | 0 | 0 | 0 | 113.749 |
| 95 | 8w-4v-improved.jpg | 0 | 0 | 0 | 0 | 34.592 |
| 96 | 8w-4v-improved.jpg | 0 | 0 | 0 | 0 | 23.385 |
| 97 | 8w-4v-improved.jpg | 0 | 0 | 0 | 0 | 51.116 |
| 98 | 8w-4v-improved.jpg | 0 | 0 | 0 | 0 | 13.392 |
| 99 | 8w-4v-improved.jpg | 0 | 0 | 0 | 0 | 26.952 |
| 100 | 8w-4v-improved.jpg | 0 | 0 | 0 | 0 | 10.081 |
| 101 | 8w-4v-improved.jpg | 0 | 0 | 0 | 0 | 135 |
| 102 | 8w-4v-improved.jpg | 0 | 0 | 0 | 0 | 126.87 |
| 103 | 8w-4v-improved.jpg | 0 | 0 | 0 | 0 | 78.366 |
| 104 | 8w-4v-improved.jpg | 0 | 0 | 0 | 0 | 138.814 |
| 105 | 8w-4v-improved.jpg | 0 | 0 | 0 | 0 | 2.899 |
| 106 | 8w-4v-improved.jpg | 0 | 0 | 0 | 0 | 125.828 |
| 107 | 8w-4v-improved.jpg | 0 | 0 | 0 | 0 | 151.113 |
| 108 | 8w-4v-improved.jpg | 0 | 0 | 0 | 0 | 137.726 |
| 109 | 8w-4v-improved.jpg | 0 | 0 | 0 | 0 | 28.61 |
| 110 | 8w-4v-improved.jpg | 0 | 0 | 0 | 0 | 59.036 |
|  |  |  |  |  | Mean | 78.35109 |
|  |  |  |  |  | Standard deviation | 53.11323 |

**Table S3.** Angular measurement of 8 wt. % - 4 V fibers.

|  | Label | Area | Mean | Min | Max | Angle |
| --- | --- | --- | --- | --- | --- | --- |
| 1 | 8 w%-4 V.tif | 0 | 0 | 0 | 0 | 19.983 |
| 2 | 8 w%-4 V.tif | 0 | 0 | 0 | 0 | 171.384 |
| 3 | 8 w%-4 V.tif | 0 | 0 | 0 | 0 | 126.87 |
| 4 | 8 w%-4 V.tif | 0 | 0 | 0 | 0 | 13.736 |
| 5 | 8 w%-4 V.tif | 0 | 0 | 0 | 0 | 59.036 |
| 6 | 8 w%-4 V.tif | 0 | 0 | 0 | 0 | 90 |
| 7 | 8 w%-4 V.tif | 0 | 0 | 0 | 0 | 36.87 |
| 8 | 8 w%-4 V.tif | 0 | 0 | 0 | 0 | 41.987 |
| 9 | 8 w%-4 V.tif | 0 | 0 | 0 | 0 | 25.201 |
| 10 | 8 w%-4 V.tif | 0 | 0 | 0 | 0 | 45 |
| 11 | 8 w%-4 V.tif | 0 | 0 | 0 | 0 | 170.538 |
| 12 | 8 w%-4 V.tif | 0 | 0 | 0 | 0 | 149.036 |
| 13 | 8 w%-4 V.tif | 0 | 0 | 0 | 0 | 28.926 |
| 14 | 8 w%-4 V.tif | 0 | 0 | 0 | 0 | 117.553 |
| 15 | 8 w%-4 V.tif | 0 | 0 | 0 | 0 | 141.34 |
| 16 | 8 w%-4 V.tif | 0 | 0 | 0 | 0 | 119.982 |
| 17 | 8 w%-4 V.tif | 0 | 0 | 0 | 0 | 153.435 |
| 18 | 8 w%-4 V.tif | 0 | 0 | 0 | 0 | 136.397 |
| 19 | 8 w%-4 V.tif | 0 | 0 | 0 | 0 | 32.905 |
| 20 | 8 w%-4 V.tif | 0 | 0 | 0 | 0 | 61.39 |
| 21 | 8 w%-4 V.tif | 0 | 0 | 0 | 0 | 172.763 |
| 22 | 8 w%-4 V.tif | 0 | 0 | 0 | 0 | 145.176 |
| 23 | 8 w%-4 V.tif | 0 | 0 | 0 | 0 | 165.964 |
| 24 | 8 w%-4 V.tif | 0 | 0 | 0 | 0 | 28.61 |
| 25 | 8 w%-4 V.tif | 0 | 0 | 0 | 0 | 30.964 |
| 26 | 8 w%-4 V.tif | 0 | 0 | 0 | 0 | 168.69 |
| 27 | 8 w%-4 V.tif | 0 | 0 | 0 | 0 | 60.945 |
| 28 | 8 w%-4 V.tif | 0 | 0 | 0 | 0 | 16.991 |
| 29 | 8 w%-4 V.tif | 0 | 0 | 0 | 0 | 58.57 |
| 30 | 8 w%-4 V.tif | 0 | 0 | 0 | 0 | 17.879 |
| 31 | 8 w%-4 V.tif | 0 | 0 | 0 | 0 | 174.806 |
| 32 | 8 w%-4 V.tif | 0 | 0 | 0 | 0 | 78.232 |
| 33 | 8 w%-4 V.tif | 0 | 0 | 0 | 0 | 131.82 |
| 34 | 8 w%-4 V.tif | 0 | 0 | 0 | 0 | 12.095 |
| 35 | 8 w%-4 V.tif | 0 | 0 | 0 | 0 | 97.125 |
| 36 | 8 w%-4 V.tif | 0 | 0 | 0 | 0 | 50.194 |
| 37 | 8 w%-4 V.tif | 0 | 0 | 0 | 0 | 20.556 |
| 38 | 8 w%-4 V.tif | 0 | 0 | 0 | 0 | 115.56 |
| 39 | 8 w%-4 V.tif | 0 | 0 | 0 | 0 | 95.194 |
| 40 | 8 w%-4 V.tif | 0 | 0 | 0 | 0 | 131.987 |
| 41 | 8 w%-4 V.tif | 0 | 0 | 0 | 0 | 114.444 |
| 42 | 8 w%-4 V.tif | 0 | 0 | 0 | 0 | 150.642 |
| 43 | 8 w%-4 V.tif | 0 | 0 | 0 | 0 | 0 |
| 44 | 8 w%-4 V.tif | 0 | 0 | 0 | 0 | 172.304 |
| 45 | 8 w%-4 V.tif | 0 | 0 | 0 | 0 | 0 |
| 46 | 8 w%-4 V.tif | 0 | 0 | 0 | 0 | 19.799 |
| 47 | 8 w%-4 V.tif | 0 | 0 | 0 | 0 | 152.354 |
| 48 | 8 w%-4 V.tif | 0 | 0 | 0 | 0 | 131.987 |
| 49 | 8 w%-4 V.tif | 0 | 0 | 0 | 0 | 173.29 |
| 50 | 8 w%-4 V.tif | 0 | 0 | 0 | 0 | 56.31 |
| 51 | 8 w%-4 V.tif | 0 | 0 | 0 | 0 | 15.018 |
| 52 | 8 w%-4 V.tif | 0 | 0 | 0 | 0 | 5.194 |
| 53 | 8 w%-4 V.tif | 0 | 0 | 0 | 0 | 131.186 |
| 54 | 8 w%-4 V.tif | 0 | 0 | 0 | 0 | 64.983 |
| 55 | 8 w%-4 V.tif | 0 | 0 | 0 | 0 | 6.34 |
| 56 | 8 w%-4 V.tif | 0 | 0 | 0 | 0 | 0 |
| 57 | 8 w%-4 V.tif | 0 | 0 | 0 | 0 | 72.759 |
| 58 | 8 w%-4 V.tif | 0 | 0 | 0 | 0 | 17.526 |
| 59 | 8 w%-4 V.tif | 0 | 0 | 0 | 0 | 168.44 |
| 60 | 8 w%-4 V.tif | 0 | 0 | 0 | 0 | 135 |
| 61 | 8 w%-4 V.tif | 0 | 0 | 0 | 0 | 105.945 |
| 62 | 8 w%-4 V.tif | 0 | 0 | 0 | 0 | 61.699 |
| 63 | 8 w%-4 V.tif | 0 | 0 | 0 | 0 | 36.027 |
| 64 | 8 w%-4 V.tif | 0 | 0 | 0 | 0 | 54.782 |
| 65 | 8 w%-4 V.tif | 0 | 0 | 0 | 0 | 43.877 |
| 66 | 8 w%-4 V.tif | 0 | 0 | 0 | 0 | 4.667 |
| 67 | 8 w%-4 V.tif | 0 | 0 | 0 | 0 | 5.906 |
| 68 | 8 w%-4 V.tif | 0 | 0 | 0 | 0 | 12.339 |
| 69 | 8 w%-4 V.tif | 0 | 0 | 0 | 0 | 150.524 |
| 70 | 8 w%-4 V.tif | 0 | 0 | 0 | 0 | 148.57 |
| 71 | 8 w%-4 V.tif | 0 | 0 | 0 | 0 | 168.69 |
| 72 | 8 w%-4 V.tif | 0 | 0 | 0 | 0 | 159.444 |
| 73 | 8 w%-4 V.tif | 0 | 0 | 0 | 0 | 17.969 |
| 74 | 8 w%-4 V.tif | 0 | 0 | 0 | 0 | 23.199 |
| 75 | 8 w%-4 V.tif | 0 | 0 | 0 | 0 | 22.479 |
| 76 | 8 w%-4 V.tif | 0 | 0 | 0 | 0 | 156.371 |
| 77 | 8 w%-4 V.tif | 0 | 0 | 0 | 0 | 8.973 |
| 78 | 8 w%-4 V.tif | 0 | 0 | 0 | 0 | 4.667 |
| 79 | 8 w%-4 V.tif | 0 | 0 | 0 | 0 | 142.524 |
| 80 | 8 w%-4 V.tif | 0 | 0 | 0 | 0 | 170.538 |
| 81 | 8 w%-4 V.tif | 0 | 0 | 0 | 0 | 2.437 |
| 82 | 8 w%-4 V.tif | 0 | 0 | 0 | 0 | 21.161 |
| 83 | 8 w%-4 V.tif | 0 | 0 | 0 | 0 | 124.992 |
| 84 | 8 w%-4 V.tif | 0 | 0 | 0 | 0 | 53.13 |
| 85 | 8 w%-4 V.tif | 0 | 0 | 0 | 0 | 117.897 |
| 86 | 8 w%-4 V.tif | 0 | 0 | 0 | 0 | 1.193 |
| 87 | 8 w%-4 V.tif | 0 | 0 | 0 | 0 | 108.435 |
| 88 | 8 w%-4 V.tif | 0 | 0 | 0 | 0 | 4.899 |
| 89 | 8 w%-4 V.tif | 0 | 0 | 0 | 0 | 122.905 |
| 90 | 8 w%-4 V.tif | 0 | 0 | 0 | 0 | 140.711 |
| 91 | 8 w%-4 V.tif | 0 | 0 | 0 | 0 | 52.125 |
| 92 | 8 w%-4 V.tif | 0 | 0 | 0 | 0 | 113.199 |
| 93 | 8 w%-4 V.tif | 0 | 0 | 0 | 0 | 172.648 |
| 94 | 8 w%-4 V.tif | 0 | 0 | 0 | 0 | 71.565 |
| 95 | 8 w%-4 V.tif | 0 | 0 | 0 | 0 | 54.162 |
| 96 | 8 w%-4 V.tif | 0 | 0 | 0 | 0 | 110.323 |
| 97 | 8 w%-4 V.tif | 0 | 0 | 0 | 0 | 151.113 |
| 98 | 8 w%-4 V.tif | 0 | 0 | 0 | 0 | 126.87 |
| 99 | 8 w%-4 V.tif | 0 | 0 | 0 | 0 | 120.579 |
| 100 | 8 w%-4 V.tif | 0 | 0 | 0 | 0 | 56.31 |
| 101 | 8 w%-4 V.tif | 0 | 0 | 0 | 0 | 104.036 |
| 102 | 8 w%-4 V.tif | 0 | 0 | 0 | 0 | 142.125 |
| 103 | 8 w%-4 V.tif | 0 | 0 | 0 | 0 | 2.603 |
| 104 | 8 w%-4 V.tif | 0 | 0 | 0 | 0 | 119.055 |
| 105 | 8 w%-4 V.tif | 0 | 0 | 0 | 0 | 66.371 |
| 106 | 8 w%-4 V.tif | 0 | 0 | 0 | 0 | 141.843 |
| 107 | 8 w%-4 V.tif | 0 | 0 | 0 | 0 | 61.557 |
| 108 | 8 w%-4 V.tif | 0 | 0 | 0 | 0 | 39.289 |
| 109 | 8 w%-4 V.tif | 0 | 0 | 0 | 0 | 3.468 |
| 110 | 8 w%-4 V.tif | 0 | 0 | 0 | 0 | 128.157 |
|  |  |  |  |  | Mean | 83.06922 |
|  |  |  |  |  | Standard deviation | 58.79253 |

**Table S4**. Angular measurement of 8 wt. % - 5 V fibers.

|  | Label | Area | Mean | Min | Max | Angle |
| --- | --- | --- | --- | --- | --- | --- |
| 1 | 8 w%-5V.tif | 0 | 0 | 0 | 0 | 30.651 |
| 2 | 8 w%-5V.tif | 0 | 0 | 0 | 0 | 53.842 |
| 3 | 8 w%-5V.tif | 0 | 0 | 0 | 0 | 52.306 |
| 4 | 8 w%-5V.tif | 0 | 0 | 0 | 0 | 56.689 |
| 5 | 8 w%-5V.tif | 0 | 0 | 0 | 0 | 105.068 |
| 6 | 8 w%-5V.tif | 0 | 0 | 0 | 0 | 12.011 |
| 7 | 8 w%-5V.tif | 0 | 0 | 0 | 0 | 90 |
| 8 | 8 w%-5V.tif | 0 | 0 | 0 | 0 | 169.216 |
| 9 | 8 w%-5V.tif | 0 | 0 | 0 | 0 | 115.017 |
| 10 | 8 w%-5V.tif | 0 | 0 | 0 | 0 | 120.379 |
| 11 | 8 w%-5V.tif | 0 | 0 | 0 | 0 | 47.291 |
| 12 | 8 w%-5V.tif | 0 | 0 | 0 | 0 | 43.264 |
| 13 | 8 w%-5V.tif | 0 | 0 | 0 | 0 | 124.287 |
| 14 | 8 w%-5V.tif | 0 | 0 | 0 | 0 | 24.944 |
| 15 | 8 w%-5V.tif | 0 | 0 | 0 | 0 | 62.447 |
| 16 | 8 w%-5V.tif | 0 | 0 | 0 | 0 | 90 |
| 17 | 8 w%-5V.tif | 0 | 0 | 0 | 0 | 81.384 |
| 18 | 8 w%-5V.tif | 0 | 0 | 0 | 0 | 58.57 |
| 19 | 8 w%-5V.tif | 0 | 0 | 0 | 0 | 171.416 |
| 20 | 8 w%-5V.tif | 0 | 0 | 0 | 0 | 77.32 |
| 21 | 8 w%-5V.tif | 0 | 0 | 0 | 0 | 83.047 |
| 22 | 8 w%-5V.tif | 0 | 0 | 0 | 0 | 56.31 |
| 23 | 8 w%-5V.tif | 0 | 0 | 0 | 0 | 147.724 |
| 24 | 8 w%-5V.tif | 0 | 0 | 0 | 0 | 162.031 |
| 25 | 8 w%-5V.tif | 0 | 0 | 0 | 0 | 21.318 |
| 26 | 8 w%-5V.tif | 0 | 0 | 0 | 0 | 77.471 |
| 27 | 8 w%-5V.tif | 0 | 0 | 0 | 0 | 136.975 |
| 28 | 8 w%-5V.tif | 0 | 0 | 0 | 0 | 165.964 |
| 29 | 8 w%-5V.tif | 0 | 0 | 0 | 0 | 27.979 |
| 30 | 8 w%-5V.tif | 0 | 0 | 0 | 0 | 162.759 |
| 31 | 8 w%-5V.tif | 0 | 0 | 0 | 0 | 162.897 |
| 32 | 8 w%-5V.tif | 0 | 0 | 0 | 0 | 62.241 |
| 33 | 8 w%-5V.tif | 0 | 0 | 0 | 0 | 20.323 |
| 34 | 8 w%-5V.tif | 0 | 0 | 0 | 0 | 10.954 |
| 35 | 8 w%-5V.tif | 0 | 0 | 0 | 0 | 24.146 |
| 36 | 8 w%-5V.tif | 0 | 0 | 0 | 0 | 74.055 |
| 37 | 8 w%-5V.tif | 0 | 0 | 0 | 0 | 21.448 |
| 38 | 8 w%-5V.tif | 0 | 0 | 0 | 0 | 164.476 |
| 39 | 8 w%-5V.tif | 0 | 0 | 0 | 0 | 168.366 |
| 40 | 8 w%-5V.tif | 0 | 0 | 0 | 0 | 149.931 |
| 41 | 8 w%-5V.tif | 0 | 0 | 0 | 0 | 23.499 |
| 42 | 8 w%-5V.tif | 0 | 0 | 0 | 0 | 14.931 |
| 43 | 8 w%-5V.tif | 0 | 0 | 0 | 0 | 4.485 |
| 44 | 8 w%-5V.tif | 0 | 0 | 0 | 0 | 159.59 |
| 45 | 8 w%-5V.tif | 0 | 0 | 0 | 0 | 27.216 |
| 46 | 8 w%-5V.tif | 0 | 0 | 0 | 0 | 70.974 |
| 47 | 8 w%-5V.tif | 0 | 0 | 0 | 0 | 21.801 |
| 48 | 8 w%-5V.tif | 0 | 0 | 0 | 0 | 39.611 |
| 49 | 8 w%-5V.tif | 0 | 0 | 0 | 0 | 46.975 |
| 50 | 8 w%-5V.tif | 0 | 0 | 0 | 0 | 116.565 |
| 51 | 8 w%-5V.tif | 0 | 0 | 0 | 0 | 26.565 |
| 52 | 8 w%-5V.tif | 0 | 0 | 0 | 0 | 150.945 |
| 53 | 8 w%-5V.tif | 0 | 0 | 0 | 0 | 167.905 |
| 54 | 8 w%-5V.tif | 0 | 0 | 0 | 0 | 18.435 |
| 55 | 8 w%-5V.tif | 0 | 0 | 0 | 0 | 150.945 |
| 56 | 8 w%-5V.tif | 0 | 0 | 0 | 0 | 5.377 |
| 57 | 8 w%-5V.tif | 0 | 0 | 0 | 0 | 155.772 |
| 58 | 8 w%-5V.tif | 0 | 0 | 0 | 0 | 42.51 |
| 59 | 8 w%-5V.tif | 0 | 0 | 0 | 0 | 12.653 |
| 60 | 8 w%-5V.tif | 0 | 0 | 0 | 0 | 113.749 |
| 61 | 8 w%-5V.tif | 0 | 0 | 0 | 0 | 25.641 |
| 62 | 8 w%-5V.tif | 0 | 0 | 0 | 0 | 66.038 |
| 63 | 8 w%-5V.tif | 0 | 0 | 0 | 0 | 107.354 |
| 64 | 8 w%-5V.tif | 0 | 0 | 0 | 0 | 165.579 |
| 65 | 8 w%-5V.tif | 0 | 0 | 0 | 0 | 48.366 |
| 66 | 8 w%-5V.tif | 0 | 0 | 0 | 0 | 49.399 |
| 67 | 8 w%-5V.tif | 0 | 0 | 0 | 0 | 53.616 |
| 68 | 8 w%-5V.tif | 0 | 0 | 0 | 0 | 47.121 |
| 69 | 8 w%-5V.tif | 0 | 0 | 0 | 0 | 50.389 |
| 70 | 8 w%-5V.tif | 0 | 0 | 0 | 0 | 164.745 |
| 71 | 8 w%-5V.tif | 0 | 0 | 0 | 0 | 53.13 |
| 72 | 8 w%-5V.tif | 0 | 0 | 0 | 0 | 54.689 |
| 73 | 8 w%-5V.tif | 0 | 0 | 0 | 0 | 55.305 |
| 74 | 8 w%-5V.tif | 0 | 0 | 0 | 0 | 156.801 |
| 75 | 8 w%-5V.tif | 0 | 0 | 0 | 0 | 140.711 |
| 76 | 8 w%-5V.tif | 0 | 0 | 0 | 0 | 61.928 |
| 77 | 8 w%-5V.tif | 0 | 0 | 0 | 0 | 74.055 |
| 78 | 8 w%-5V.tif | 0 | 0 | 0 | 0 | 138.013 |
| 79 | 8 w%-5V.tif | 0 | 0 | 0 | 0 | 123.69 |
| 80 | 8 w%-5V.tif | 0 | 0 | 0 | 0 | 21.161 |
| 81 | 8 w%-5V.tif | 0 | 0 | 0 | 0 | 159.775 |
| 82 | 8 w%-5V.tif | 0 | 0 | 0 | 0 | 149.3 |
| 83 | 8 w%-5V.tif | 0 | 0 | 0 | 0 | 9.162 |
| 84 | 8 w%-5V.tif | 0 | 0 | 0 | 0 | 171.254 |
| 85 | 8 w%-5V.tif | 0 | 0 | 0 | 0 | 62.65 |
| 86 | 8 w%-5V.tif | 0 | 0 | 0 | 0 | 66.801 |
| 87 | 8 w%-5V.tif | 0 | 0 | 0 | 0 | 83.29 |
| 88 | 8 w%-5V.tif | 0 | 0 | 0 | 0 | 99.462 |
| 89 | 8 w%-5V.tif | 0 | 0 | 0 | 0 | 53.746 |
| 90 | 8 w%-5V.tif | 0 | 0 | 0 | 0 | 6.34 |
| 91 | 8 w%-5V.tif | 0 | 0 | 0 | 0 | 39.56 |
| 92 | 8 w%-5V.tif | 0 | 0 | 0 | 0 | 54.162 |
| 93 | 8 w%-5V.tif | 0 | 0 | 0 | 0 | 30.069 |
| 94 | 8 w%-5V.tif | 0 | 0 | 0 | 0 | 51.34 |
| 95 | 8 w%-5V.tif | 0 | 0 | 0 | 0 | 11.592 |
| 96 | 8 w%-5V.tif | 0 | 0 | 0 | 0 | 45 |
| 97 | 8 w%-5V.tif | 0 | 0 | 0 | 0 | 56.31 |
| 98 | 8 w%-5V.tif | 0 | 0 | 0 | 0 | 53.13 |
| 99 | 8 w%-5V.tif | 0 | 0 | 0 | 0 | 67.62 |
| 100 | 8 w%-5V.tif | 0 | 0 | 0 | 0 | 151.39 |
| 101 | 8 w%-5V.tif | 0 | 0 | 0 | 0 | 90 |
| 102 | 8 w%-5V.tif | 0 | 0 | 0 | 0 | 160.56 |
| 103 | 8 w%-5V.tif | 0 | 0 | 0 | 0 | 122.471 |
| 104 | 8 w%-5V.tif | 0 | 0 | 0 | 0 | 98.531 |
| 105 | 8 w%-5V.tif | 0 | 0 | 0 | 0 | 20.556 |
| 106 | 8 w%-5V.tif | 0 | 0 | 0 | 0 | 85.601 |
| 107 | 8 w%-5V.tif | 0 | 0 | 0 | 0 | 0 |
| 108 | 8 w%-5V.tif | 0 | 0 | 0 | 0 | 138.814 |
| 109 | 8 w%-5V.tif | 0 | 0 | 0 | 0 | 105.945 |
| 110 | 8 w%-5V.tif | 0 | 0 | 0 | 0 | 56.31 |
|  |  |  |  |  | Mean | 80.14084 |
|  |  |  |  |  | Standard deviation | 52.80223 |
